# Students' motivation for rubric use in the EFL classroom assessment environment

**DOI:** 10.3389/fpsyg.2022.895952

**Published:** 2022-07-25

**Authors:** Chunxiu He, Jiayan Zeng, Jianlin Chen

**Affiliations:** ^1^School of English Studies, Shanghai International Studies University, Shanghai, China; ^2^School of Education, Shanghai International Studies University, Shanghai, China; ^3^China Center for Language Planning and Policy Studies, Shanghai International Studies University, Shanghai, China

**Keywords:** rubric use, effort, task motivation, trait motivation, EFL classroom assessment, assessment for learning

## Abstract

The effectiveness of a rubric depends on how it is enacted. Although students' efforts in rubric use vary, few studies have investigated the hidden motivations when rubrics are utilized for classroom assessment. This qualitative study attempts to categorize students' effort in rubric use and identify personal differences and contextual factors influencing the effort in the EFL classroom assessment environment. A total of 79 students at a Chinese university participated in the study. The data collected included their classroom oral presentation results and nine case study informants' retrospective interviews on their processes of rubric use. Focuses were drawn upon students' perceptions and practices of rubric use throughout the task process. Totally, three types of effort patterns emerged in light of students' self-ratings and descriptions of the use. The intense kind held firm trust in rubric utility and thus utilized the rubric to develop the targeted competence throughout the whole process. The medium type either selectively followed the rubric in optional phases of the process due to their judgments of the rubric and the task. The loose type was least responsive to the rubric since their actions seemed largely affected by their self-efficacy and prior experience. Results showed that students' effort in rubric use in classroom assessment was the outcome of cognitive appraisals of a rubric, students themselves, and a task. The study highlights trait motivation and task motivation in the effectiveness of rubric use in assessment practices. Implications on rubric employment and task design are drawn to tap students' motivation for rubric use to achieve assessment for learning.

## Introduction

Rubrics are widely used in both summative and formative assessments at different education levels (Reddy, 2007; Brookhart and Chen, 2015) and in a range of disciplines in higher education (Reddy and Andrade, 2010). Although rubrics could be flexible in format and content in practice (Dawson, 2015), typical rubrics are embedded with three essential features of rubrics, that is, evaluative criteria, matching quality definitions, and a scoring strategy (Popham, 1997). Assessment criteria like rubrics could enable teachers to make justifiable evaluations (Popham, 1997; Andrade, 2000; Panadero and Jonsson, 2013) and help students understand the desired performance and make an improvement (Andrade and Du, 2005; Panadero and Jonsson, 2013; Wu et al., 2021) and thus bear evaluative and instructional value (Popham, 1997; Andrade, 2005) and contribute in the paradigm of assessment for learning (Black and Wiliam, 2009; Zhou and Deneen, 2016).

In language teaching and learning, rubrics are particularly important instruments for classroom performance tasks such as speaking and writing (Lane and Tierney, 2008; Sadler, 2009; Wang, 2017) since rubrics could promote the alignment between task design and curriculum objectives (Zhou and Wang, 2019) and the development of students' integrated skills (Popham, 1997). However, the promise does not come along with the launch of rubrics since they might feature “the good” and “the bad” and “the ugly” depending on “how they are created and how they are used” ((Andrade, 2005), p. 27). The effectiveness of rubric use may at worst go null if students disregard the rubric for an assessment task (e.g., Hafner and Hafner, 2003; Andrade and Du, 2005). Thus, it is of significance to understand students' effort in rubric use for a particular task, that is, how students devote their efforts to rubric use and what urges them to do so. The unique situation of students “is integrated into the task at hand” (Bearman and Ajjawi, 2019, p. 3). Understanding students' motivation for rubric use for a particular task could provide insights into “how to successfully implement the use of rubrics for formative purposes” (Panadero and Jonsson, 2013, p. 142) and facilitate assessment for learning. Hence, the present study aims to explore students' effort in rubric use and illuminate the factors that may motivate (or demotivate) the effort in the use in the EFL classroom assessment environment. Specifically, it addresses two questions: (1) How do students report their efforts in rubric use in an oral presentation task? (2) What motivational factors moderate students' effort in rubric use in the EFL classroom assessment environment?

## Literature review

### Students' effort in rubric use

The immediate purpose of rubric use is to facilitate assessment and improve performance (Popham, 1997), and the long-term goal is to promote sustained learning (Bearman and Ajjawi, 2019). Sustained engagement with rubrics has to be committed for better performance when students treat rubrics as references in the self-regulatory process and activate self-assessment as a learning strategy (Andrade, 2001; Panadero and Alonso-Tapia, 2013). Rubrics could promote meaning-making, coordinate sustained learning, and develop reflective knowing when students receive rubrics as invitations to activity (Bearman and Ajjawi, 2019) and become active participants in the learning process to get aligned with “the dominant educational ethos” of assessment for learning (Davison, 2019, p. 439).

To steer assessment for learning, rubrics should become materials and utilized fully as instructions, goals, and memos throughout the process of task implementation for recursive planning, implementation, and evaluation (Zimmerman and Moylan, 2009; Panadero and Alonso-Tapia, 2013; Wang, 2017). In practice, it exists that rubrics are utilized by students in the classroom to the extremes: unfathomable worship regardless of a mismatch in their understanding and teacher expectations (Andrade and Du, 2005), and overt neglect in instructional situations (Schafer et al., 2001; Lim, 2013; Jonsson, 2014). For those students, rubrics are either criteria compliance (Sadler, 2007) or of limited instructional value (Lim, 2013). But the reality is not clear-cut yet, that is, how students utilize rubrics and what they are considering in the process remain afloat. Given the importance of rubrics in supporting students' active learning, it is worthwhile to probe into students' rubric use.

### Factors for students' rubric use

A couple of factors have been identified to moderate students' rubric use and are categorized into with-in rubric factors and rubric-user factors (Wang, 2017). With-in rubric factors are design features of rubrics, such as language, content/coverage/criteria, structure, descriptors, and score range (Reddy and Andrade, 2010; Jonsson, 2014; Wang, 2017). These are matters of construct validity of a rubric because any assessment form needs to accurately and consistently assess what it intends to evaluate (Reddy and Andrade, 2010).

Rubric–user factors refer to student characteristics, among which learners' domain knowledge of the assessed skill, length of intervention, and learner profiles like educational level and gender have been discussed (e.g., (Panadero and Jonsson, 2013; Wang, 2017)). However, motivation on students' rubric use is not sufficiently expounded (Panadero and Jonsson, 2013). In general, motivation determines students' effort in rubric use and influences the performance quality. For instance, (Reddy and Andrade, 2010) investigated the function of rubrics in directing students' motivation and effort toward performance enhancement in two different sets of students. It found that the rubric developed initially for one set of students motivated toward higher pay or a better job did not bring about the required effort and quality of responses from the other set of students with a short-term goal of passing a course. Thus, the rubric had to be revised to include it for the appropriate use by both sets. In other cases, students might be fettered by criteria and act in compliance when they are overconcerned with the presumed expectations (e.g., Boud and Falchikov, 2006; Sadler, 2007; Torrance, 2007; Zhou and Deneen, 2016). Yet, how personal differences, such as goal orientation, prior experience, and academic performance, shape students' motivation that directs their effort in rubric use, remains largely unknown (Panadero and Jonsson, 2013).

In addition, contextual factors, or analogically named rubric-used factors, are found to interfere with the effects of rubric use (Green and Bowser, 2006), and thus, a rubric has to be adapted to the situated context (Reddy and Andrade, 2010). In Green and Bowser (2006), the same rubric encountered validity issues when being used by two groups of students who were either concluding their literature reviews or just beginning the literature review process for the master's thesis in two universities. Hamp-Lyons (2016) draws on Broad (2003) argument that many traditional rubrics are problematic “because of their lack of contextual relevance and failure to grow organically from contexts and purposes” (p. A2). Context-bound studies of rubric use are necessary to identify any pattern or draw any conclusions and propagate its utility in diverse contexts (Reddy and Andrade, 2010; Panadero and Jonsson, 2013). Effort and motivational considerations upon rubric utilization would bring close attention to the person and the context involved.

### Effort and motivation in the classroom assessment environment

A classroom assessment environment is defined as a context in which particular assessments occur, and it is set up by assessment-related factors (Brookhart et al., 2006). The classroom assessment environment is viewed as a sociocultural reality experienced and interpreted by individuals, and learners' internal thoughts and feelings form part of that experience (Brookhart, 1997).

Effort refers to post-decisional commitment, including willful persistence and adaptive strategy use (Corno, 1993; Brookhart et al., 2006). Efforts could be mental and behavioral endeavors guided by motivational factors (Brookhart et al., 2006). Motivation, although a complex and multifaceted construct, is defined as a disposition toward something in educational psychology (Brookhart et al., 2006). In the classroom context, motivation plays a main role in controlling and directing an activity or a task (Julkunen, 2001). Motivation could be distinguished according to the task and learner, that is, *motivation as a state* or *task motivation* to refer to situation-specific motivation, and *motivation as a trait* or *trait motivation* with a general orientation in learners (c.f. Boekaerts, 1987; Julkunen, 2001; Mozgalina, 2015).

When it comes to a situated context, task motivation projects the importance of the characteristics of a task (Julkunen, 2001). An individual's task motivation is regarded as ‘the composite dynamic outcome of a complex range of contextual influences as well as learner internal factors and the intrinsic properties of the task’ (Dörnyei and Ushioda, 2011, p. 60). Since sustained task engagement provides students with more opportunities to interact and thus learn the language, it is important for researchers and teachers to find out task features that can enhance students' task motivation and, as a consequence, language learning (Mozgalina, 2015). Task motivation should be optimally studied in three stages: the initial stage, the actual performance stage, and the evaluation stage (Boekaerts, 1987; Julkunen, 1989). Learners' cognitive appraisals of tasks and encounters regulate the choice of appropriate strategies and the effort expenditure on a task (Julkunen, 2001). Task motivation is found to vary depending on factors such as students' attitudes toward a task, student characteristics, and the relationship between academic achievement and students' affective response (Dornyei, 2001; Julkunen, 2001).

In terms of trait motivation, assessment-related motivational factors cluster into three categories of student characteristics: learners' general learning disposition (self-concept as a learner), task-specific attitude (interest and enthusiasm), and task-specific learning disposition (goal orientation and self-efficacy) (Harlen and Crick, 2003). Keller (1983, 1994) formulates four determinants of motivation that influence an individual's degree of effort that he/she will exert in learning: interest, relevance, confidence, and outcomes. Learners generally deviate in two types of goal orientations connected to the task such as performance orientation and mastery orientation (Ames and Archer, 1988). Performance orientation highlights the accomplishment of the learning task, while mastery orientation emphasizes the mastery of skills and improvement of abilities. Bandura (1997) connects confidence with outcomes in self-efficacy, which is defined as “beliefs in one's capabilities to organize and execute the courses of action required to produce given attainments” (p. 3). Self-efficacy is found to be an element of language learning motivation and positively relates to effort and performance (Brookhart et al., 2006; Kormos et al., 2011).

Given students' divergent attitudes toward rubrics, the complex factors that may influence their practices, and the significance of motivation in controlling effort, it is necessary to gain insights into students' effort patterns and motivational factors in rubric use in the ongoing classroom assessment environment.

## The study

The study takes oral presentation tasks in the EFL classroom assessment environment for discussion as oral presentations are a prevalent mode of activity and assessment in tertiary settings across the globe (Tsang, 2018), and presenting is universally acknowledged as an essential qualification of highly educated graduates (van Ginkel et al., 2017). The research questions for this study are formulated to feature the source of the major data (Maxwell, 2012) as follows: (1) How do students report their efforts in rubric use in an oral presentation task? (2) What motivational factors moderate students' effort in rubric use in the EFL classroom assessment environment? To address the questions, in-depth semi-structured interviews serve as the major approach as qualitative exploratory investigations of learners' self-reports could be employed to retrieve perspectives on motivated behavior (Dörnyei and Ushioda, 2011). No intervention is imposed on the instructional design of the course as real classroom practices should be the domains of field studies for classroom assessment, although the range of quality levels may vary to a large extent (Brookhart et al., 2006).

### Context and participants

The sampling pool was from a class of 79 senior students at a top language-featured university in China. The course students attended was entitled *Comprehensive English*, by which students could earn credits by accomplishing assessment tasks for the enhancement of language skills and proficiency. Specifically, the students were required to accomplish five formative assessment tasks, accounting for 50% of the final score for the course, composed of two oral English presentations—one written English book reviews and two English writings (each 10%), and a summative assessment task of a final test constituting another half of the final score. As for the presentation tasks related to this study, the first was on a self-select topic, and the second was a book review from a book list offered by the instructor. For this, two analytical task-specific rubrics in English were created on the Internet (http://rubistar.4teachers.org); (www.teach-nology.com) by the instructor and posted in the class group on WeChat (a popular social network service in China) when the tasks were assigned, with a caution that the performances would be evaluated accordingly and questions regarding the rubrics would be welcomed anytime. The focus of our study, the second rubric for the book review report, consisted of 10 categories: *posture and eye contact, speaks clearly, preparedness, content, enthusiasm, vocabulary, stay on topic and understanding, volume, knowledge base*, and *critical thinking*, each having four descriptor levels (see Appendix). The schedule to perform the tasks was negotiated between the students and the tutor. The students performed the tasks individually, and their performances were video recorded by their classmates. Meanwhile, two raters (the first author and the instructor) independently marked on the copies of the rubrics with written comments and returned oral feedback to the students by referring to the rubrics, the scores being withheld. To check the quality of the rubrics, inter-rater reliability was measured in the quality processes (Johnson et al., 2000), with the first presentation task *r*_rater1−rater2_ = 0.734 (*p* < 0.05, *n* = 79) and the second *r*_rater1−rater2_ = 0.850 (*p* < 0.05, *n* = 79).

Purposive sampling was used to screen participants, and gender, on-stage performance, and performance results were attended to, for the purpose of balance and heterogeneity. Totally, 10 (five male and five female) students were contacted to participate in the in-depth semi-structured interviews. Except that one female student quit before the interview, nine students (from five provinces/municipalities) joined the one-on-one interviews coordinated by the first author. Anonymous names were assigned to the participants in the report of the study to protect privacy. The nine informants' profiles and performances are summarized in Table 1.

**Table 1:** Learner profiles and performances of the participants.

**Student**	**Gender**	**Age**	**Major**	**Self-rating/effort pattern**	**Performance achievement**			
					**Presentation 1**		**Presentation 2**	
					**Rater 1**	**Rater 2**	**Rater 1**	**Rater 2**
Julie	F	21	French	^*^/intense	9	9.5	9.2	9
Laurie	M	22	Arabic	^*^/intense	9	9.6	9.5	9.7
Philip	M	22	Arabic	4/medium	8.5	9.8	9	9.4
Brian	M	21	Vietnamese	5/medium	8.8	8.5	7.5	7.5
Tim	M	23	Italian	7/medium	8	9	8.5	9
Kelly	F	22	Korean	9/intense	8.5	9	7.5	8.2
Sue	F	22	Spanish	4/loose	7.4	8.5	8	7.4
Ruth	F	22	Portuguese	^*^/loose	7.5	6	7	7.2
Gary	M	21	Italian	7/medium	7.5	7.5	8	7.5

* *means students did not give a self-rating score*.

### Data collection and analysis

The interviews were conducted at the end of the semester in January 2020 soon after the students had finished the course. Before the interview, the participants were explicitly informed of the research purpose and the fact that no alteration to the scores would incur. In the interviews, the participants were reminded to focus on their rubric use for the second presentation task since they could recall more clearly owing to the closer due date (c.f. Gass and Mackey, 2000). The stimulated questions centered on students' opinions on oral presentation competence and the tasks, their perceptions of rubrics, detailed descriptions of their use of the second rubric, and their considerations in the process. The participants were also required to give a score out of 10 to measure their effort in the rubric use. Each interview lasted about 30–50 min. Interview audios, video recordings of the presentations, and assessment results of all the tasks in the semester by the whole class were collected to provide triangulation for qualitative inquiry (c.f. Miles et al., 1994; Maxwell, 2012).

All of the audiotaped interviews (in L1/Chinese) were transcribed verbatim (63,523 Chinese characters in total) and double-checked. Maximal fidelity was pursued with care to the transcriptions of the opinions of the participants, and the participants were contacted through WeChat for confirmation and clarification in case of unclear points. The data were checked and analyzed through an abductive thematic analysis in response to epistemology and research questions (Patton, 2015). For the purpose of trustworthiness and credibility, the overall data analysis was recursively crosschecked by the authors, and suggestions were sought from two qualitative research experts in language assessment and education. Categories and themes were settled after a sequential and iterative procedure, ending in three a priori categories of *rubric utility, trait motivation*, and *task motivation*, highlighting students' perceived rubric utility, student characteristics, and perceived task features, respectively. An example is presented in Table 2 to illustrate how the data were analyzed.

**Table 2:** Steps of data analysis.

**Steps**	**Actions taken**	**Examples**
1st	Coding in detail	Interest toward the task form Interest toward the topic
2nd	Grouping codes and naming basic themes	Interest toward the task
3rd	Identifying illustrative excerpts	“I did the tasks more out of interest, because I had got a job commitment and the score was meaningless to me. It's like that if I do a project out of interest, I would like to spend more time on it.”
4th	Identifying key themes	“Interest” under “task features”
5th	Joining key themes under overarching themes	“Task features” under “task motivation”

## Findings

In this section, the findings are presented on students' perceptions of rubrics and practices of rubric use. Effort patterns are summarized based on the analysis of students' reports of the utilization processes. Motivation for rubric use is illustrated in the categories of rubric utility, trait motivation, and task motivation.

### Effort patterns of students' rubric use

Students' self-rating scores were first referenced, among which 4 and 7 were tentatively taken as the dividing lines of effort patterns. Students' self-reports were then checked iteratively to modify the classification, and three effort patterns emerged in terms of two rules: whether students followed all of the criteria and whether they utilized the rubric in the whole process of preparation, performance, and after-thought. Totally, two students (Sue and Ruth) indicated that they seldom referred to the rubric, and they formed the loose effort group; four students (Philip, Brian, Tim, and Gary) from the medium effort group admitted that they made use of the rubric selectively, attempting to meet some of the criteria and follow the rubric in either one or two phases of the process. In total, three students (Julie, Laurie, and Kelly) fell into the intense effort group since they attended to all the criteria throughout the whole process. For instance, Kelly explained that she cautiously prepared her speech according to the rubric, recalled some of the criteria during the performance, and checked her performance against the rubric afterward.

Kelly: I read the rubric to understand the requirements before the preparation. I tried to adhere to all the requirements such as *stay on topic* in the drafting. When I finished my drafting, I checked it against the rubric to see whether I had gone astray. Then when I stood on the platform, I consciously made more eye contact with the audience as required. I also recalled the rubric in my mind when I watched the video, having an eye on fluency and postures.

It is noteworthy that mastery-oriented students (to be expounded later) from both the intense and the medium effort groups like Julie, Laurie, Philip, and Brian overtly explained that they did not pay equal attention to the criteria but approached them selectively by focusing on the ones that they valued. For instance, Laurie said that he was mainly concerned about the requirement for the inclusion of strength and weakness in *critical thinking* since in his perspective, the two were task-specific for a book review, whereas others were general tasks for oral presentations.

Laurie: In *critical thinking*, the rubric mentioned strengths and weaknesses. A hit for structure, I thought it was. I didn't do well at that when I began to write my draft. (In the draft) I mentioned something not so good (about the book), for instance, I said there were some defects in the book but it was excellent as a whole, and then I explained some good points, but in the next part, I returned to its weak points. Later, I searched online for some (information) on how to do critical thinking and how to write an academic article. I also took some online classes, which highlighted logical order in academic writing or structure in this sense. I realized that the order in my draft was weakness first and strength afterward. So I changed the order.

Similarly, Philip also browsed online to include related information for criteria *content, knowledge base*, and *critical thinking*. However, unlike Julie and Laurie who went back to the rubric after the performance, Philip admitted that he did not utilize the rubric during and after the performance.

Philip: The rubric was effective. For instance, it required us to demonstrate knowledge base, so I searched on the Internet to gain more understanding of the book…. I used some criteria for preparation, especially I structured my speech according to the criteria such as *stay on topic and understanding*, and the descriptor on background understanding pushed me to make more preparations…but I did not care about the rubric during and after the performance either.

For the medium effort group, students did not believe that rubrics could convey the teacher's expectations fully. They thought that there must be something extra behind the evaluation of an individualized performance.

Philip: There were indeed some basics in the rubrics. Still, rubrics couldn't entail all the components for the skill measurement. So I didn't dance to the tune.

Brian: Even with the rubric, I was not sure about the teacher's criteria. The scoring must be subjective because the rubric was the same for all the students.

For the loose effort group, students tended to deal with the task according to their ingrained criteria developed from their previous experience, and the performances were mainly based on their knowledge about the assigned topics and English proficiency.

Sue: We had done many presentations before, and thus it was more likely that we just followed the old routine.

Ruth: The rubrics didn't confine me, nor help me. I simply did not think they affect me, good or bad, not too much. I just wrote what I wanted to write for the presentations. I seldom referred to the rubrics.

In summary, students made unleveled endeavors in rubric use. The intense effort group held a respectful attitude toward the rubric and valued it throughout the whole process. The medium effort group showed reservations about the rubric and utilized it partially. The loose effort group was least responsive to the rubric and relied mainly on their previous experience and personal judgments. It could be inferred that rubric use is the end of complex cognitive processes.

### Motivation for rubric use

**Figure 1:**
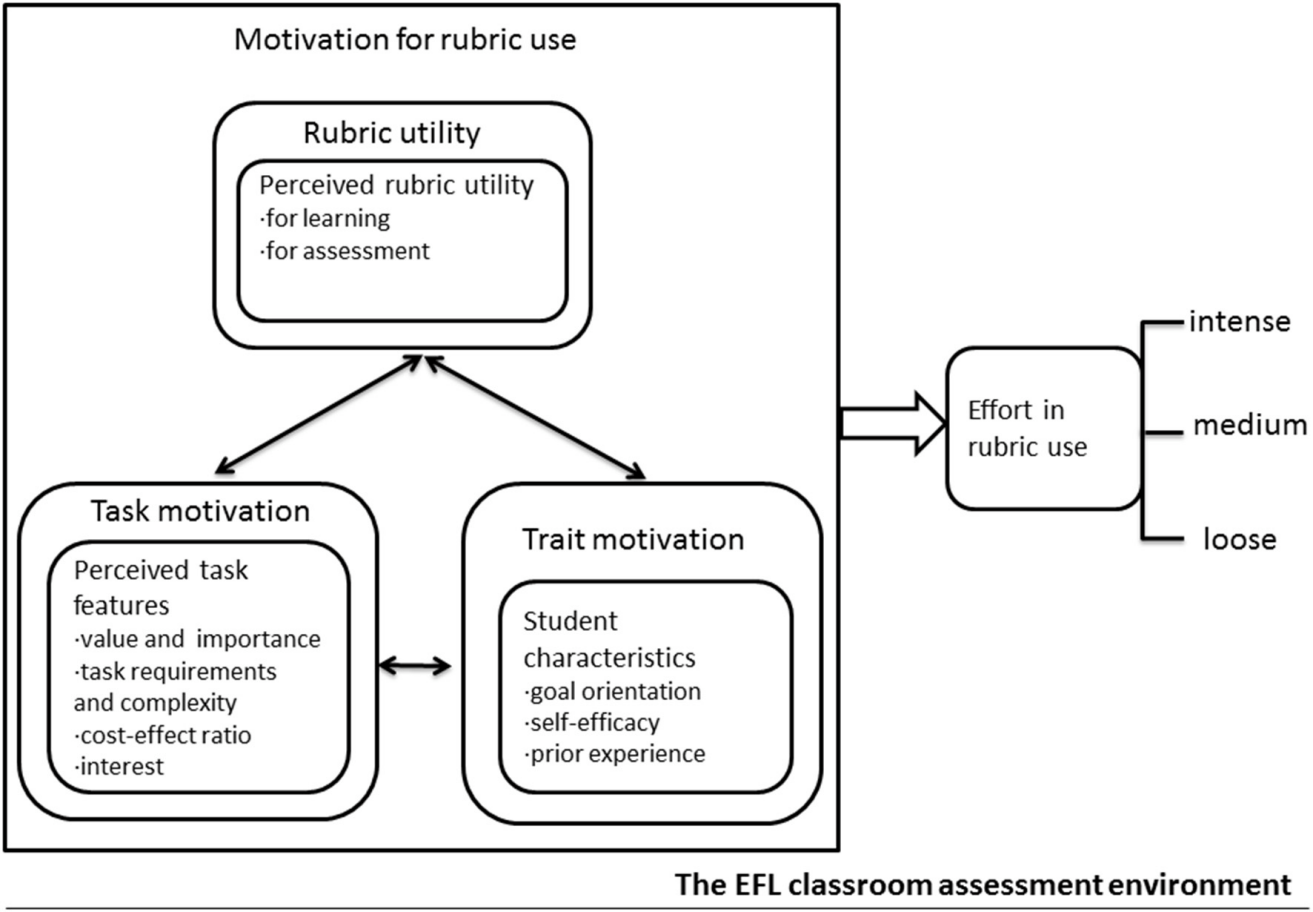
Students' motivation for rubric use in the EFL classroom assessment environment: A data-driven model.

Iterative analysis of the interviews found that students' motivation for rubric use in the EFL classroom assessment environment involves students' cognitive appraisals of rubrics, students themselves, and tasks. Hence, motivational factors are delineated into three overarching categories and subcategories (Figure 1): (1) rubric utility highlighting students' perceived rubric utility for learning and assessment; (2) trait motivation identifying student characteristics manifested in goal orientations, self-efficacy, and prior experience; and (3) task motivation related to students' perceived task features reflected in the value and importance of a task weighed against task requirements and complexity, cost-to-effect ratio, and personal interest in the task.

#### Perceived rubric utility

In general, rubrics were acknowledged as an effective learning guide by the students. Most of the participants held that rubrics explicitly convey requirements in a comprehensive framework and thus are of use to task implementation. For instance, Kelly from the intense effort group had a high opinion of the rubric.

Kelly: The rubric listed the criteria from many aspects, i.e., requirements for English public speaking. We could prepare to the point and in advance….If we prepared carefully, I believe all of us could live up to the teacher's expectations.

Students from both the medium and loose effort groups did not think a rubric could fully entail the elements of public speaking, and they wanted to present something personal.

Brian: The rubric was quite comprehensive, but there was still a lot depending on impromptu performance, different forms, such as a picture. Some students were quite professional, for instance, they used many technical terms and included videos, songs, and others, something to your surprise. Those were not listed in the rubric, but audiences always look forward to novel expressions.

On the other hand, although the students agreed that the use of rubrics could lead to better performances and enhance presentation skills, they held reserved opinions on the evaluative role of the rubric. Uncertainty toward objectivity and fairness was the source of doubts.

Gary: We didn't know how the teacher would apply rubrics, because rubrics are expressed in words and words are arbitrary. For instance, as for “speak clearly,” how to measure it must be personal. Therefore, the scoring is subjective.

Rubrics as unitary measurement standards were doubted whether they could fairly assess individualized performances.

Sue: I was not sure about the teacher's real intention, as rubrics are the same for all students.

To sum up, acknowledgment of the instructional value of rubrics convinced the students to take rubrics as references for task performance. Both the intense and medium effort groups believed the rubric could help them perform, but the latter seemed to act beyond the rubric and intended to construct the response with personal understanding, for instance, to impress with personality. It seemed that uncertainty toward the evaluative value refrained the medium and loose effort groups from identifying rubrics as trustworthy standards and led to increased personal understanding and judgments.

#### Student characteristics

In the study, goal orientations, self-efficacy, and prior experience were found to be salient student characteristics in underpinning trait motivation for rubric use.

1) Goal orientations

Students' goals deviated into performance orientation and mastery orientation in the study. Totally, three of the nine participants (Julie, Laurie, and Philip) confided their long-term goals of language learning and aspired to master public speaking competence. For instance, both Laurie and Julie were planning to further their education in the United Kingdom.

Julie: I always stress the improvement of oral presentation competence. I think I can express myself naturally and calmly (in public). My goal in foreign language learning is to communicate freely in public. Actually, I am planning to study in the UK.

Similarly, Philip articulated that he was going to practice English speaking in the approaching winter vacation as his prospective job involved international negotiation. Brian, although did not utter any long-term language learning goals, claimed that the firm belief in the competence and intense interest in the task drove him to devote himself to the task; thus, he was also mastery-orientated. Other students did not relate any specific goal for the skill enhancement, although also agreed on the importance of the competence. The tasks, in their perspective, were simply assignments from the teacher. They were more performance-oriented and consequently exhibited passiveness in task implementation.

Kelly: If there were no tasks, I would not initiate to improve my English.

The two kinds of goal orientations directed distinct self-regulation strategies in rubric use. Different from mastery-oriented students' endeavor to further their understanding of the criteria, performance-oriented students tended to focus on accessible requirements but circumvented far-reaching ones in their pursuit of scores.

Tim: I didn't spare much concern on it (critical thinking), but just skipped it. It was too difficult to prepare, you know, but the score equaled with others.

These examples showed that goal orientation posed an important motivational variable for students' attitudes toward the tasks and directed their self-regulation strategies. It was noteworthy that students with mastery-oriented goals tended to go deep into the rubric to understand the criteria better. In addition, student characteristics such as self-efficacy and prior experience also moderated students' effort in rubric use.

2) Self-efficacy

Self-efficacy varies in students' belief in their capability. In all, three participants (Ruth, Julie, and Brian) in the study were typical in their self-reports of self-efficacy. Ruth's low self-efficacy made her ignore the rubric. She confessed that she felt incapable of making use of rubrics.

Ruth: The guiding function of rubrics was apparent, but I doubted I was able to apply them. Rubrics didn't help me much, because I could not satisfy the criteria at all. I knew they were there and I wish I could fulfill them, but I just could not make it.

Conversely, both Julie and Brian were quite conceited with their public speaking competence. Because of their high self-efficacy, they prepared for the tasks by incorporating the criteria into their understanding of a good speech.

Julie: My performance was quite natural, not timid. My mindset was quite balanced…I just took a look at the rubric once in a while but did not remember the criteria clearly. During the preparation and when I finished drafting, I resorted to the rubric to check and make some supplements.

Brian: I kept the rubric in mind, having a general idea about what the teacher expected from us and attempting to realize it. But I did not try to satisfy all the criteria.… Instead, I deliberately imitated some excellent or successful speeches.

It can be seen that self-efficacy is a salient factor in influencing students' effort in rubric use and might dominate their self-regulation strategies in case of extremes of high and low.

3) Prior experience

It was noticeable that the students reported limited encounters with detailed written rubrics, although they had a plentiful experience of being evaluated and assessed during their school years.

Ruth: Teachers seldom offered us written rubrics ahead of tasks…. They might vocalize emphatically in the classroom, things like to perform naturally or to offer more eye contact.

The students formed opinions on rubrics based on experience and acted accordingly. For instance, Sue disclosed that her class was asked to use a task-specific rubric for peer assessment in a former language learning class, but the rubric was not seriously treated because “*the task did not count much*.” Philip recalled his experience in a project design competition, in which the judges rated the submitted projects according to a rubric. In his opinion, rubrics for oral presentation tasks were more liable to personal bias because the performances “*depend more on the audience's spontaneous feeling*.”

Furthermore, unpleasant prior experiences may offset students' efforts. The fact that the endeavor that Brain invested in a previous task not paid off affected his attitude toward similar tasks in a negative way.

Brian: Last semester, I worked much harder on a presentation task. But later on I found what I carefully prepared, for instance, what I prepared for 1 day or half a day, did not make any difference from that, by my classmate just for 10 min. I don't think scores matched efforts.

Similarly, new experience with rubrics could accumulate to foster or recast students' perceptions, just as Kelly delineated:

When I finished the task, I saw that the teacher evaluated our performance strictly according to the rubric. It enhanced my knowledge about rubrics.

To sum up, students struck a balance between the rubric and their knowledge developed from the previous experience. Simultaneously, new experiences continued to develop the knowledge subtly.

#### Perceived task features

In the study, task value and importance, task requirements and complexity, cost-to-effect ratio, and interest emerged as subthemes of perceived task features. First and foremost, students commonly agreed that oral presentation competence was integral to study and career.

Julie: Public speaking skill is, English public speaking in particular, on one hand, a component of language competence, and on the other hand, a test of logic and expression. Besides, expressing in a foreign language is different from that in Chinese, and more challenging for sure. In my opinion, enhancing the ability to express in English is very important for study and work.

Since students valued public speaking competence, oral presentation tasks were regarded as opportunities to practice the skill.

Sue: English, thought as the most important foreign language, needs practicing. We need a large amount of input to keep up the level, or else there would be landslides. The tasks were opportunities to push me to improve my English.

In addition, concerns about task requirements and complexity impacted rubric use. Students might skip some of the criteria if the requirements were too complicated.

Philip: The rubric contained too many pea-sized bits, and each category had four levels…. I made a balance between what I wanted to present and the requirements in the criteria. After all, one presentation took up only 10 points.

When requirements were perceived as too high to reach, perceived inaccessibility discouraged students from attempting.

Ruth: It is like the teacher gives you a perfect MA thesis and asks you to imitate it, but students would not trouble to read it. Anyway, if I can't reach it, I give up.

Moreover, the importance of a task was measured against the cost-to-effect ratio in the total score. Totally, five students admitted that scoring was critical in deciding their effort for the tasks and rubrics.

Gary: It was mainly an urge for the score, which was related to the grade point in the final. Scores are very important to students, and we would put in effort for the sake of scores.

Sue: The score would change something. You see, a rubric is a standard. If the ratio had gone down, we would have performed at will; if it had been higher, surely we would have abided by the rubrics more closely.

Students confessed that they would feel pitiful for a demanding task with limited prospective rewards. A low ratio would have impaired students' seriousness toward the task and the course.

Philip: I might prepare as hard, but the ratio would have impacted my attitude toward the course. Rational or irrational (the ratio was), but I had to take it.

Conversely, a high ratio might render an extra burden on students because that might pose a threat to the grade point average.

Tim: To do an oral book review is the most difficult of all the assessment tasks, and it would be fine if it counted the most in the final score. But in that case, it might pose a challenge for us to pass the course.

Students weighed the cost that the tasks might trigger in the whole picture of study loads. Alternative occupations might drag them away from the task if they valued others much more.

Gary: I didn't attempt hard to meet the criteria in the rubrics because I had to prepare for my postgraduate entrance examination. I had some concerns about energy and time.

By contrast, interest in an assessment task could somewhat offset the demands the task imposed. For instance, Tim admitted that he would have dealt with the task casually in case no option was offered to students. Brian confided that his motivation for the task mainly came from interest.

Brian: I did the tasks more out of interest because I had got a job commitment and the score was meaningless to me. It's like that if I do a project out of interest I would be happy to spend more time on it.

It can be summarized that task value and importance counted for all students, and performance-oriented students were more easily impacted by task requirements and complexity and cost-to-effect ratio, for which interest served as a lubricant.

## Discussion

Through retrospective interviews on an oral presentation task, this study stratifies students' effort in rubric use and indicates that the effort is the outcome of students' cognitive appraisals of a rubric, themselves, and a task. Compared with Chinese EFL learners' unitary adoption of a rubric as an instructional tool throughout the task process in the literature (Wang, 2017), the study presents a pluralizing picture of effort patterns and strategies in rubric use. The study highlights trait motivation and task motivation in the effectiveness of rubric use in the EFL classroom setting (e.g., Hafner and Hafner, 2003; Panadero and Romero, 2014; Wang, 2017).

### Understanding students' effort in rubric use for formative assessment

Findings from this study extend the present understanding of students as key rubric users for formative assessment (Stiggins, 2001). Although students' comments confirmed many of the arguments made to advocate the adoption of a rubric for formative classroom assessment, students' efforts in rubric use and strategies employed varied to a large extent. Ultimately, two rules were developed according to students' self-reports of the rubric use: whether students carefully followed all the criteria and whether they applied the rubric throughout the process of preparation, performance, and after-thought. In terms of the rules and students' self-rating scores, three groups of intense, medium, and loose effort patterns emerged. Students might treat the rubric carefully throughout the whole process of the task out of different motives and fall into the intense effort group. Students with performance orientation in the group might act in the way of “honor” and “uncritical acceptance” of the criteria (Andrade and Du, 2005, p. 7), but students with mastery orientation endeavored to deepen their understanding of the focused criteria by expanding information sources, which confirms students undertake “invitational enactments” in rubric use, and rubrics promote learning while inviting students into a “productive space” (Bearman and Ajjawi, 2019, p. 1). The medium effort group applied the criteria partially and selectively. Their efforts in rubric use relied on the judgment regarding the task and the rubric. When students harbored disagreement with the rubric, for instance, “too trivial” in Philip's comment, they chose to enact the criteria to emphasize what they valued. Hence, both the behaviors of the intense effort group with mastery orientation and the medium effort group challenge the claim that rubrics lead to criteria compliance and constrained learning experience (e.g., Boud and Falchikov, 2006; Sadler, 2007; Torrance, 2007). As for the loose effort group, however, students tended to put the rubric aside and act according to their long built-up understanding, resulting in the limited instructional value of a rubric (Lim, 2013).

Our study indicates that student characteristics are salient motivational factors in determining the strategies in rubric use, particularly goals and self-efficacy. It echoes that variables like “personal goals, including goal commitment, and self-efficacy are often, although not invariably, the most immediate, conscious motivational determinants of action” (Locke and Latham, 2002, p. 709). In the study, mastery-oriented students and performance-oriented students approached the rubric with different strategies out of distinct motives. The former like Laurie and Philip regarded the task as an opportunity to work on their oral competence, and the rubric functioned to invite them to extend their understanding through online learning. Conversely, the latter selected the criteria with an instrumental mindset, that is, to skip the difficult ones and work for the easy ones, and their efforts into the rubric were apt to fluctuate due to contextual factors such as task features. This suggests teachers should help students set up mastery-oriented goals of language learning to stimulate learner agency (c.f. Murphy, 1996). In addition, it evidenced that perceived self-efficacy and prior experience also impact students' decisions in rubric use. For instance, low-self-efficacy students like Ruth in the study overtly expressed her lack of self-confidence in complying with the rubrics owing to her unsatisfactory prior experience with similar tasks and thus performed free from the rubric. By contrast, high-self-efficacy students like Julie and Brian were determined about what constituted an excellent speech and acted confidently on the podium. The result corroborates previous studies that self-efficacy built upon prior experience is important in student confidence and performance (Pintrich and Schrauben, 1992; Brookhart et al., 2006). It also extends that extreme self-efficacy might reduce students' effort in rubric use in that it might devalue a rubric. Hence, self-efficacy should be observed in students to ensure rational rubric utilization. On the one hand, individuals with low self-efficacy need to be encouraged through persuasive communication in the possibility to attain the goal and strategy provision to facilitate the attainment (Locke and Latham, 2002), for which following rubrics/assessment criteria is a convenient and effective one. On the other hand, individuals with high self-efficacy need to be reminded of the value of a rubric in introducing reflection and creativity (Bearman and Ajjawi, 2019).

### Encouraging explicit rubric use in the EFL classroom assessment environment

The study indicates that in the EFL context, opportunities to encounter explicit assessment criteria are insufficient to equip students with assessment literacy. For one thing, rubrics were deemed to be an effective instructional guide to task implementation and skill enhancement. For another, the evaluative value of rubrics was not harbored by students due to their doubts about the assessment process. This corroborates that students lack sustained exposure to explicit task-specific rubrics and knowledge of apt rubric use (Schafer et al., 2001; Lim, 2013) because teachers like Chinese tertiary language teachers displayed a preference for non-achievement criteria and regarded assessment criteria as teachers' tacit knowledge (Zhou and Deneen, 2016; Zhou and Wang, 2019). Nevertheless, students in the study understood that rubrics were the convergence of teachers' expectations and pathways to quality products. This challenges the claim that students appear to have little understanding of a connection between the teachers' expectation and “a broader definition of quality” (Andrade and Du, 2005, p. 7).

The literature endorses utilizing transparent and specific achievement-related criteria to construct a trustworthy classroom assessment environment to enhance student learning (Brookhart et al., 2006; Wiliam, 2010) in the paradigm of assessment for learning. Contrary to that, explicit assessment criteria were found to be commonly concealed from the participants from five provincial areas of the country during their school years and even the university. This rings a caution for the development of teacher assessment literacy and the scrutiny of teacher training programs (Wu et al., 2021), but the discussion is beyond the scope of our study. In this case, the study is incongruous with the opinion that students proceeding to higher educational levels are in less need of training and explanations for rubric use (Panadero and Jonsson, 2013; Jonsson, 2014). We claim that orientation to rubric use is necessary for all occasions of rubric-embedded assessment practices in learning settings. Dialogical interpretation of assessment criteria is helpful for students before embarking on a task. Students need to be encouraged to actively get involved in the assessment process (Sadler, 2009; Wu et al., 2021) including the design and use of explicit assessment criteria (Matshedisho, 2020). Assessment literacy, belief in rubric utility in particular, should be enhanced for students similar to those in the study.

### Linking task features with student characteristics in task design

The study reveals the interplay between task features and student characteristics in shaping students' incentives for rubric use. In addition to the aforementioned nexus between the strategies and student characteristics, it underlines the influence of task features on students' attitudes toward a task and a rubric in formative assessment and confirms that “small decisions in task design can have a subtle but important influence on students' motivation” and behavior when it comes to a classroom setting (Mozgalina, 2015, p. 130). Contrary to the claim that constrained choice conditions are more beneficial for task motivation and task engagement (Mozgalina, 2015), freedom to choose topics was appreciated as empowerment of personal interest, which might be owing to the different language levels that the participants were in: advanced and intermediate in this study vs. beginners in Mozgalina's. For beginners, ego depletion (c.f. Vohs et al., 2008, 2010) is more hazardous in that all acts of choice or self-control increase cognitive burdens and resource depletion. The comparison suggests that the higher language proficiency learners possess, the more autonomy they should be entitled to.

Instrumentalism that is found common in high-stake tests was also prominent in students' appraisals of task requirements and complexity and cost-to-effect ratio, and dominated self-regulation strategies in performance-oriented students, which echoes that specific decisions regarding task difficulty like topic, content, and format are related to task motivation and performance (Julkunen, 2001; Locke and Latham, 2002) and extends the influential factors to include the cost-to-effect ratio and interest. Consequently, task design should take student characteristics into account. For instance, task difficulty conveyed through the requirements in a rubric needs to be set tangible, and the cost-to-effect ratio of a task should be rationally controlled to mobilize learner agency. In sum, task design should feature students' concerns and demands to stimulate their task motivation as formative assessment tasks need to be carefully adopted to counterbalance the influence of summative assessment, particularly in grading-emphasized cultural settings (Carless, 2011; Wang, 2017).

## Conclusion

This study contributes to the existing understanding of students' effort in rubric use by conducting a contextual analysis of tertiary students' perceptions and practices of rubric utilization in a local EFL learning context. It provides empirical support to illuminate effort patterns and motivation in rubric use. The study finds that students' effort in rubric use is the end of cognitive appraisals of a rubric, students themselves, and a task. These findings have practical implications for rubric employment and task design in classroom assessment to boost learner agency in utilizing a rubric in the paradigm of assessment for learning.

It should be noted that a limit regarding generalization is inevitable for an interpretative study with a small sample. Furthermore, the study uses a cognitive approach to motivation and centers on learners themselves, but it does not claim that motivation is immune to social contexts such as teachers and peers. Future research is warranted to study deeper into the present factors and expand others by adopting diversified methodologies and recruiting larger samples in similar or different contexts.

## Data availability statement

The original contributions presented in the study are included in the article/Supplementary material, further inquiries can be directed to the corresponding author/s.

## Ethics statement

Ethical review and approval was not required for the study on human participants in accordance with the local legislation and institutional requirements. Written informed consent for participation was not required for this study in accordance with the national legislation and the institutional requirements.

## Author contributions

CH: conceptualization, methodology, investigation, data analysis, data collection, writing–original draft, and review and editing. JZ: conceptualization, methodology, data collection, and data analysis. JC: conceptualization, funding acquisition, review and editing, and supervision. All authors contributed to the article and approved the submitted version.

## Funding

This work was supported by the Supervisor-led Scholarship Program from Shanghai International Studies University (Grant No. 2020114207), Shanghai, China, obtained by JC.

## Conflict of interest

The authors declare that the research was conducted in the absence of any commercial or financial relationships that could be construed as a potential conflict of interest.

## Publisher's note

All claims expressed in this article are solely those of the authors and do not necessarily represent those of their affiliated organizations, or those of the publisher, the editors and the reviewers. Any product that may be evaluated in this article, or claim that may be made by its manufacturer, is not guaranteed or endorsed by the publisher.
