# Supplementary material for: Students' motivation for rubric use in the EFL classroom assessment environment
Source: Front Psychol. 2022 Jul 25;13:895952. doi: 10.3389/fpsyg.2022.895952 (PMC9358141; doi:10.3389/fpsyg.2022.895952)
Supplement: Supplementary file 1 [file Data_Sheet_1.PDF]

RubiStar Rubric Made Using:
RubiStar (http://rubistar.4teachers.org)

Oral Presentation Rubric : Oral Report

Teacher Name:

Student Name: ____

CATEGORY	4	3	2	1
**Posture and Eye Contact**	Stands up straight, looks relaxed and confident. Establishes eye contact with everyone in the room during the presentation.	Stands up straight and establishes eye contact with everyone in the room during the presentation.	Sometimes stands up straight and establishes eye contact.	Slouches and/or does not look at people during the presentation.
**Speaks Clearly**	Speaks clearly and distinctly all (100-95%) the time, and mispronounces no words.	Speaks clearly and distinctly all (100-95%) the time, but mispronounces one word.	Speaks clearly and distinctly most (94-85%) of the time. Mispronounces no more than one word.	Often mumbles or can not be understood OR mispronounces more than one word.
**Preparedness**	Student is completely prepared and has obviously rehearsed.	Student seems pretty prepared but might have needed a couple more rehearsals.	The student is somewhat prepared, but it is clear that rehearsal was lacking.	Student does not seem at all prepared to present.
**Content**	Shows a full understanding of the topic.	Shows a good understanding of the topic.	Shows a good understanding of parts of the topic.	Does not seem to understand the topic very well.
**Enthusiasm**	Facial expressions and body language generate a strong interest and enthusiasm about the topic in others.	Facial expressions and body language sometimes generate a strong interest and enthusiasm about the topic in others.	Facial expressions and body language are used to try to generate enthusiasm, but seem somewhat faked.	Very little use of facial expressions or body language. Did not generate much interest in topic being presented.
**Vocabulary**	Uses vocabulary appropriate for the audience. Extends audience vocabulary by defining words that might be new to most of the audience.	Uses vocabulary appropriate for the audience. Includes 1-2 words that might be new to most of the audience, but does not define them.	Uses vocabulary appropriate for the audience. Does not include any vocabulary that might be new to the audience.	Uses several (5 or more) words or phrases that are not understood by the audience.
**Stay on Topic and Understanding**	Stays on topic all (100%) of the time Presenter has a clear understanding of the material presented.	Stays on topic most (99-90%) of the time.Presenter has a 80-90% understanding of the material presented.	Stays on topic some (89%-75%) of the time.Presenter has a 70-79% understanding of the material presented.	It was hard to tell what the topic was.Presenter has a poor understanding of the material presented.
**Volume**	Volume is loud enough to be heard by all audience members throughout the presentation.	Volume is loud enough to be heard by all audience members at least 90% of the time.	Volume is loud enough to be heard by all audience members at least 80% of the time.	Volume often too soft to be heard by all audience members.

**Knowledge Base**	Proper background information on the topic was given. Enough essential information given to allow the audience to effectively to evaluate the topic.	90% background information on the topic was given. 90% essential information given to allow the audience to effectively to evaluate the topic.	80% background information on the topic was given. 80% essential information given to allow the audience to effectively to evaluate the topic.	Background information on the topic was not given. Essential information was not given to allow the audience to effectively to evaluate the topic.
**Critical Thinking**	The main issues, the strengths, weaknesses, and conclusions in this area are clearly identified. Theoretical positions, empirical evidence, recommendations are presented.	90% of the main issues, the strengths, weaknesses, and conclusions in this area are clearly identified. Theoretical positions, empirical evidence, recommendations are presented.	70% of the main issues, the strengths, weaknesses, and conclusions in this area are clearly identified. Theoretical positions, empirical evidence, recommendations are presented.	The main issues, the strengths, weaknesses, and conclusions in this area are poorly identified. Theoretical positions, empirical evidence, recommendations are poorly presented.

Date Created: **Nov 05, 2019 03:23 am (CST)**

Copyright © 2000-2007 Advanced Learning Technologies in Education Consortia ALTEC

To view information about the Privacy Policies and the Terms of Use, please go to the following web address:
http://rubistar.4teachers.org/index.php?screen=TermsOfUse
