# Supplementary material for: Students' motivation for rubric use in the EFL classroom assessment environment
Source: Front Psychol. 2022 Jul 25;13:895952. doi: 10.3389/fpsyg.2022.895952 (PMC9358141; doi:10.3389/fpsyg.2022.895952)
Supplement: Supplementary file 2 [file Data_Sheet_2.PDF]

Table 1 Learner profiles of the participants

Student	Gender	Age	Major	Self-evaluation of reference to the rubric	Performance achievement levels
S1	F	21	French	Medium	Advanced
S2	M	22	Arabian	Medium	Advanced
S3	M	22	Arabic	Low	Advanced
S4	M	21	Vietnamese	Medium	Intermediate
S5	M	23	Italian	Medium	Intermediate
S6	F	22	Korean	High	Intermediate
S7	F	22	Spanish	Low	Intermediate
S8	F	22	Portuguese	Low	Low
S9	M	21	Italian	Medium	Low

Table 2 An example of a student's interpretation of a descriptor

Top level descriptor in 'Critical Thinking' in the rubric for book review report	An excerpt from the transcription of S3's book review report on *The Prophet* (Kahlil Gibron)
The main issues, the strengths, weaknesses, and conclusions in this area are clearly identified.<br>Theoretical positions, empirical evidence, recommendations are presented.	Just to use a little piece of critical thinking, we will find what Gibran depicted is not similar to how the real world runs at all. In addition to this, there are two very important parts in the book that he didn't give any proof or explanation. One is from the chapter on love, and one is from the chapter on beauty.
